# The Neuroprotective Effects of Primary Functional Components Mulberry Leaf Extract in Diabetes-Induced Oxidative Stress and Inflammation

**DOI:** 10.1021/acs.jafc.4c09422

**Published:** 2025-02-02

**Authors:** Zi-Xiang Lin, Chau-Jong Wang, Hsin-Wei Tu, Meng-Ting Tsai, Meng-Hsun Yu, Hui-Pei Huang

**Affiliations:** † School of Medicine, Chung Shan Medical University, Taichung 40201, Taiwan; ‡ Department of Health Industry Technology Management, Chung Shan Medical University, Taichung 40201, Taiwan; § Department of Medical Research, Chung Shan Medical University Hospital, Taichung 40201, Taiwan; ∥ Ministry of Health and Welfare, Shuang-Ho Hospital, New Taipei City 23561, Taiwan; ⊥ Institute of Medicine, Chung Shan Medical University, Taichung 40201, Taiwan; # Department of Nutrition, Chung Shan Medical University Hospital, Taichung 40201, Taiwan; ∇ Department of Nutrition, Chung Shan Medical University, Taichung 40201, Taiwan; ○ Department of Biochemistry, School of Medicine, Chung Shan Medical University, Taichung 40201, Taiwan

**Keywords:** diabetes, neurodegeneration, mulberry leaf extract, chlorogenic acid, neochlorogenic acid

## Abstract

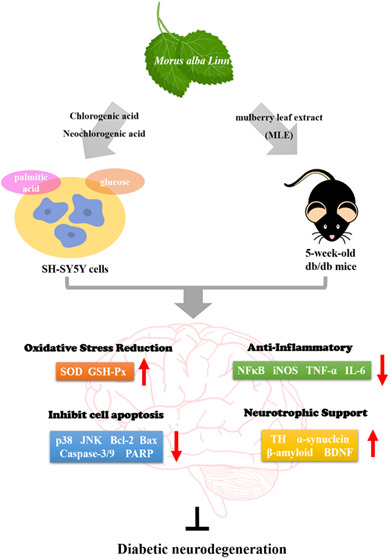

Diabetes-associated neurodegeneration may result from increased oxidative stress in the brain under hyperglycemic conditions, which leads to neuronal cell death. The current study employs the neuroblastoma cell line SH-SY5Y and db/db mouse model of diabetes maintained on a high-fat diet to investigate the neuroprotective effects of the primary functional components of mulberry (Morus alba Linn) leaf extract (MLE), chlorogenic acid (CGA), and neochlorogenic acid (NCGA). CGA and NCGA demonstrated the ability to enhance the activities of the antioxidant enzymes superoxide dismutase and glutathione peroxidase, and attenuate inflammation via regulating nuclear factor erythroid 2-related factor 2 (Nrf2), nuclear factor-κB (NFκB), and inflammatory cytokines, thereby protecting SH-SY5Y cells from oxidative damage induced by palmitic acid and high glucose. CGA and NCGA were found to decrease the expression of proinflammatory proteins α-synuclein and amyloid-β (Aβ). In addition, CGA and NCGA treatments increased the expression of tyrosine hydroxylase (TH) and brain-derived neurotrophic factor (BDNF). Furthermore, MLE supplementation in the animal model resulted in decreased levels of α-synuclein and Aβ concomitant with an elevated expression of TH. These experimental findings suggest that the neuroprotective effects of CGA and NCGA may be mediated via three pathways: reducing oxidative stress, decreasing neuronal inflammation, and enhancing BDNF expression.

## Introduction

Diabetes mellitus (DM) is a chronic metabolic disorder characterized by high levels of blood glucose resulting from insufficient insulin production or insulin resistance.^1^ The metabolic disorder arising from the consumption of a high-fat and high-glucose diet stimulates mitochondrial dysfunction and decreases antioxidant responses in neurons. The disorder is generally recognized as a significant contributor to the accelerated progression of neurodegeneration in patients with DM.^2^ Excessive accumulation of reactive oxygen species (ROS) leads to the production of inflammatory cytokines such as tumor necrosis factor α (TNF-α) and interleukin 6 (IL-6) mediated by the neuroinflammation-related transcription factor nuclear factor-κB (NFκB), ultimately resulting in the initiation of apoptosis in the neural cells.^3^

Dopaminergic neurons in the substantia nigra are responsible for releasing dopamine and modulating the activity of the corpus striatum, a region that plays a critical role in the regulation of voluntary movements.^4,5^ These dopaminergic neurons are particularly susceptible to oxidative stress.^6^ In fact, a strong association has been demonstrated between oxidative stress and the pathogenesis of neurodegenerative diseases such as Alzheimer’s disease (AD) and Parkinson’s disease (PD). In PD, ROS can interfere with the proapoptotic and antiapoptotic proteins of the B-cell lymphoma 2 (Bcl2) family, facilitating the elevated expression of initiator (caspases 8, 9, and 10) and effector (caspases 3 and 7) caspases, ultimately causing neuronal apoptosis. Morphological shrinkage and DNA fragmentation discovered in the neurons of the brain in patients with PD also support the aforementioned mechanism.^7^ Additionally, the pathophysiology of AD involves the oxidative stress-driven hyperphosphorylation of the protein Tau, which interrupts the conduction of nerve impulses and leads to c-Jun N-terminal kinase (JNK)-mediated apoptosis and cell-cycle disruption.^8^ Thus, apoptosis plays a pivotal role in the pathophysiology of AD and PD. Furthermore, the detrimental effects of a rapid increase in ROS/reactive nitrogen species and the ensuing inflammatory response in neurodegenerative diseases have been previously demonstrated. A poor modulation of the crosstalk between nuclear factor erythroid 2-related factor 2 (Nrf2) and NFκB triggered by ROS can result in the upregulation of the proinflammatory cytokines. The ensuing chronic inflammation resulting from oxidative stress underlies the pathogenesis of AD and PD.^8,9^ Consequently, supplementation with antioxidants has been proposed to protect dopaminergic neurons from oxidative damage, thereby mitigating the decline in motor function and physical activity associated with DM.

Leaves of mulberry (*Morus alba* Linn) are widely recognized for their health benefits and commonly consumed as herbal tea to aid in the reduction of blood pressure and the levels of blood sugar as well as cholesterol.^10,11^ Various bioactive compounds, particularly the phenolic acids, such as protocatechuic, cryptochlorogenic acid (CCGA), chlorogenic (CGA), and neochlorogenic acids (NCGA), have been shown to be abundantly present in mulberry leaf extract (MLE). In our laboratory’s previous analysis of mulberry leaves, we found that the yield of MLE was 32%, while the contents of CGA and NCGA were 0.238 and 0.355%, respectively.^12,13^ CGA has been shown to enhance glucose tolerance and insulin sensitivity, which are critical aspects in the management of metabolic disorders such as diabetes.^14^ Additionally, CGA has been found to reduce lipid accumulation in the liver and improve overall lipid profiles by modulating the expression of key metabolic enzymes and receptors, including the peroxisome proliferator-activated receptor-α (PPAR-α).^15^ NCGA, a less-studied isomer of CGA, has also demonstrated antioxidant and anti-inflammatory effects.^16−19^ These findings underscore the therapeutic potential of CGA and NCGA in the management of metabolic diseases including DM and its many complications. While CGA and NCGA are the major functional components in MLE, their potential to ameliorate neuronal death in patients with diabetes has not been fully explored. This study employs two systems, the db/db mice (a model for type 2 diabetes) and the human neuroblastoma cell line SH-SY5Y, to elucidate the efficacy and mechanisms underlying the activity of MLE and its active components, CGA and NCGA; the roles of the antioxidant and anti-inflammatory properties of MLE and its components in preventing diabetes-induced neuronal death in the db/db mice were also investigated.

## Materials and Methods

### Materials and Reagents

Dulbecco’s modified Eagle’s medium–high-glucose (DMEM/H), Dulbecco’s phosphate-buffered saline (PBS), ethylenediaminetetraacetic acid (EDTA)-trypsin, and fetal bovine serum (FBS) were obtained from Gibco/BRL (Gaithersburg, MD). d-Glucose, *N*-acetyl-l-cysteine, l-glutamine, palmitic acid (PA), sodium bicarbonate, CGA, antimouse IgG-peroxidase antibody, and antirabbit IgG-peroxidase antibody were obtained from Sigma-Aldrich (St. Louis, MO). 3,3′-Diaminobenzidine (DAB) chromogen, DAB copper, DAB hydrogen peroxide, DAB inhibitor, and horseradish peroxidase (HRP) multimer were obtained from Ventana Medical Systems (Tucson, AZ). NCGA was obtained from ChemFaces Biochemical (Hubei, China). Paraformaldehyde was acquired from PanReac AppliChem (Darmstadt, Germany). Carboxylated H2DCFDA analogue (carboxy-H2DCFDA, C400) was purchased from Thermo Fisher Scientific (Lenexa, KS).

### MLE

*M. alba* Linn, the white mulberry, was used in this study. Dried mulberry leaves (100 g) were boiled in 3000 mL of double-distilled water, cooled to room temperature, and stored in a refrigerator overnight. The MLE was lyophilized and frozen at −20 °C until further use.

### 1,1-Diphenyl-2-picrylhydrazil (DPPH) Radical Scavenging Assay

CGA, NCGA, CCGA, and MLE (0.00025% solutions) were used as the test samples, and 0.00025% *N*-acetyl-l-cysteine (NAC, an antioxidant and glutathione precursor), purchased from Sigma-Aldrich (St. Louis, MO), was used as the positive control. The test samples (50 μL) were added to the wells of a 96-well plate. DPPH (0.4 mM, 150 μL) was added to the test samples. After incubation in the dark for 30 min, the absorbance of the samples was measured at 517 nm (*A*_517_). The scavenging rate was calculated as follows: Scavenging rate (I%) = [1 – (*A*_517_ of sample/*A*_517_ of control)] × 100%

### Cell Culture

The human neuroblastoma cell line SH-SY5Y (CRL-2266; ATCC) was cultured in DMEM/H supplemented with 10% FBS, 5% antibiotic-antimycotic solution, 2 mM l-glutamine, 0.1 mM nonessential amino acids, 1 mM sodium pyruvate, and 3.7 g/L sodium bicarbonate at 37 °C in a humidified atmosphere with 5% carbon dioxide (CO_2_). The medium was changed every other day, and the cells were subcultured when 80% confluence was attained.

### Estimation of Cell Viability

The SH-SY5Y cells were seeded onto the wells of a 96-well plate at a density of 2.5 × 10^4^ cells/mL in 200 μL of medium per well and cultured in an incubator for 24 h. The test components CGA and NCGA (0, 1, 4, 10, and 40 μM), glucose (0, 25, 50, 100, and 200 mM), and PA (0, 0.1, 0.2, 0.3, and 0.5 mM) were added to the cells. After incubation for 24 h, the MTT reagent (0.5 mg/mL) was prepared and added to each well. The plate was then incubated at 37 °C for 3 h to allow formation of formazan crystals. The crystals were then dissolved in 2-propanol, and the absorbance was measured at 563 nm.

### Estimation of Intracellular ROS

2 ×10^4^ of cells were seeded onto a 24-well plate containing 50 mM glucose and 0.3 mM PA and treated with CGA (40 μM) or NCGA (40 μM) for 24 h. The treated cells were stained with carboxylated H_2_DCFDA analog (carboxy-H_2_DCFDA, C400) for 1 h. Subsequently, the cells were incubated in the dark for 45 min and centrifuged at 130*g* for 5 min. Following the removal of the supernatant, the content of ROS was measured by flow cytometry using the BD FACSCalibur Flow Cytometer (BD Biosciences) in accordance with the manufacturer’s instructions.

### Western Blot Analysis

In the Western blot analysis, the SH-SY5Y cells were seeded onto the wells of a 10 cm dish at a density of 1 × 10^5^ cells/mL and treated with components for the indicated time duration. After the treatment, the cells were lysed by adding radioimmunoprecipitation assay (RIPA) buffer containing 1% NP-40 (CAS 9016–45–9), 50 mM Tris-base (CAS 77–86–1), 0.1% SDS (CAS 151–21–3), 0.5% deoxycholic acid (CAS 83–44–3), 150 mM NaCl (pH 7.5), and 1.7 μg/mL leupeptin (CAS 103476–89–7). The total protein content of the samples was quantified using the Bradford protein assay. The protein samples were then subjected to sodium dodecyl sulfate polyacrylamide gel electrophoresis (SDS-PAGE), and the separated proteins were subsequently transferred onto a poly(vinylidene difluoride) (PVDF) membrane. The membrane was incubated with the primary antibody at 4 °C overnight. Subsequent incubation with a horseradish peroxidase-labeled secondary antibody allowed the visualization of the bands using ECL detection reagents and a luminescent image analyzer (LAS 4000, Fujifilm, Japan). ImageJ software was utilized for measuring the band intensity, with β-actin serving as the loading control.

### Staining of Cells with 4′,6-Diamidino-2-phenylindole (DAPI)

Apoptosis was evaluated using DAPI in accordance with the manufacturer’s instructions. After 24 h of treatment as previously described, the cells were incubated with DAPI for 1 h at 37 °C. Images were captured using a fluorescence microscope (Nikon DIAPHOT-300, Tokyo, Japan).

### Annexin V/Propidium Iodide (PI) Staining for Flow Cytometry Analysis

The Dead Cell Apoptosis Kit with Annexin V for Flow Cytometry (Invitrogen) was used for assessing apoptosis in accordance with the manufacturer’s protocol. Briefly, the cells were seeded onto the wells of a 10 cm dish at a density of 1 × 10^5^ cells/mL and treated with components and incubated at 37 °C for 24 h in the presence of 5% CO_2_. The cells were detached via EDTA-trypsin treatment and centrifuged for 5 min. The supernatant was discarded, and the cells were stained with fluorescein isothiocyanate (FITC)-labeled Annexin V and PI for 15 min. Subsequently, 400 μL of 1× annexin-binding buffer was added. The stained cells were analyzed using the BD FACSCalibur Flow Cytometer (BD Biosciences).

### Animals and Experimental Design

The experimental animals were used according to the previous study.^20^ The db/db (BKS.Cg-*Dock7*^m^ + /+ *Lepr*^db^/JNarl) and C57BL/6JNarl (control) mice aged 4–5 weeks were provided by the National Laboratory Animal Center (NLAC), NARLabs, Taiwan. The animal study was approved by the Institutional Animal Care and Use Committee of the Chung Shan Medical University (IACUC, CSMU), Taiwan (approval number 2025), and the recommendations outlined in the Guide for the Care and Use of Laboratory Animals from the National Institutes of Health (USA) were followed. The mice were housed in a controlled environment at 25 °C ± 1 °C with 55 ± 5% humidity and a 12 h light/dark cycle. After 1 week of acclimatization, the mice were randomly assigned to four groups (*n* = 3 per group), including the control, db/db, HFD, and MLE groups. The control and db/db groups were fed a normal diet, while the HFD and MLE groups were provided with an HFD containing 20% lard oil and 1.25% cholesterol. The MLE group received an additional daily dose of MLE at 250 mg/kg via oral gavage.

### Sacrifice and Sampling of Mice

The mice were sacrificed after a 12-week feeding period. Anesthesia was induced using CO_2_, followed by transcardiac perfusion with PBS until a clear outflow was observed. Subsequently, the mice were decapitated, and the brain was removed following perfusion with 4% paraformaldehyde at 4 °C for 24 h. The fixed brain was then preserved in 30% sucrose. For the PBS-perfused brain, both the midbrain and corpus striatum were dissected and removed. The dissected tissues were stored at −80 °C.

### Blood Serum Analysis

Blood samples were collected via cardiac puncture during sacrifice and transferred to blood collection tubes. The samples were then centrifuged at 3000*g* for 10 min, and the sera collected for the estimation of aspartate aminotransferase (AST), alanine aminotransferase (ALT), cholesterol, triglycerides (TG), low-density lipoprotein cholesterol (LDL-C), high-density lipoprotein cholesterol (HDL-C), uric acid (UA), creatinine (CRE), blood urea nitrogen (BUN), hemoglobin A1c (HbA1c), and insulin were measured using Beckman Coulter AU680 chemistry analyzer (Brea, CA). The measurements were done in accordance with the manufacturer’s protocol.

### Immunohistochemistry (IHC) Analysis

The tissue samples were stained with primary antibodies against tyrosine hydroxylase (TH), α-synuclein, and amyloid-β (Aβ) at 37 °C for 1 h. After being washed with PBS containing 0.5% Triton X-100 (PBST) and treated with HRP multimer for 20 min, the sections were again rinsed with PBST and stained with DAB. The images were visualized using a Nikon Eclipse E600 microscope (Nikon Instruments).

### Statistical Analysis

All quantitative data have been presented as the mean ± the standard deviation (SD) from triplicate samples in three independent experiments. SigmaPlot version 12.5 software (SJC, CA) was used for analyzing the data. Student’s *t*-test was used to compare the differences between the two groups. A *p*-value of <0.05 was considered statistically significant.

## Results

### Estimation of the Antioxidant Capacity of MLE and Its Major Components

**Figure 1:**
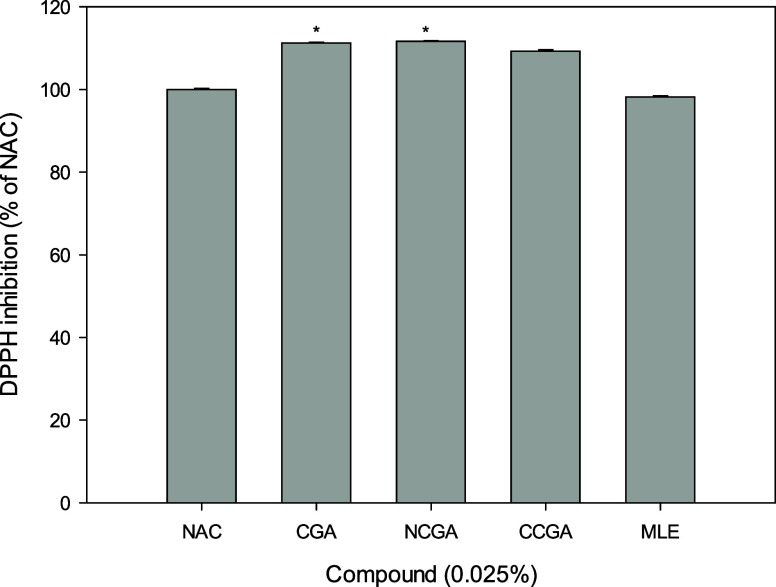
DPPH radical scavenging assay using compounds extracted from MLE. The antioxidant capacity of the compounds in MLE was evaluated herein, with NAC as the positive control set to 100%. Data from triplicate experiments are expressed as mean ± SD.

The DPPH radical scavenging assay was used for estimating the antioxidant capacity of MLE and its major compounds CGA, NCGA, and CCGA. The results revealed that the DPPH scavenging activities of CGA, NCGA, and CCGA were higher than those of equivalent concentrations of NAC. The scavenging abilities of CGA and NCGA were approximately 11% higher than that of NAC, representing a statistically significant difference, while that of CCGA was 9% higher; however, the scavenging activity of MLE was slightly lower than that of NAC (by 2%, Figure 1), suggesting that CGA and NCGA have strong antioxidant properties and are the main antioxidants in MLE. Therefore, CGA and NCGA were selected for subsequent cell-based experiments.

### CGA and NCGA Mitigate PA- and High-Glucose (HG)-Induced Cell Death in SH-SY5Y Cells

**Figure 2:**
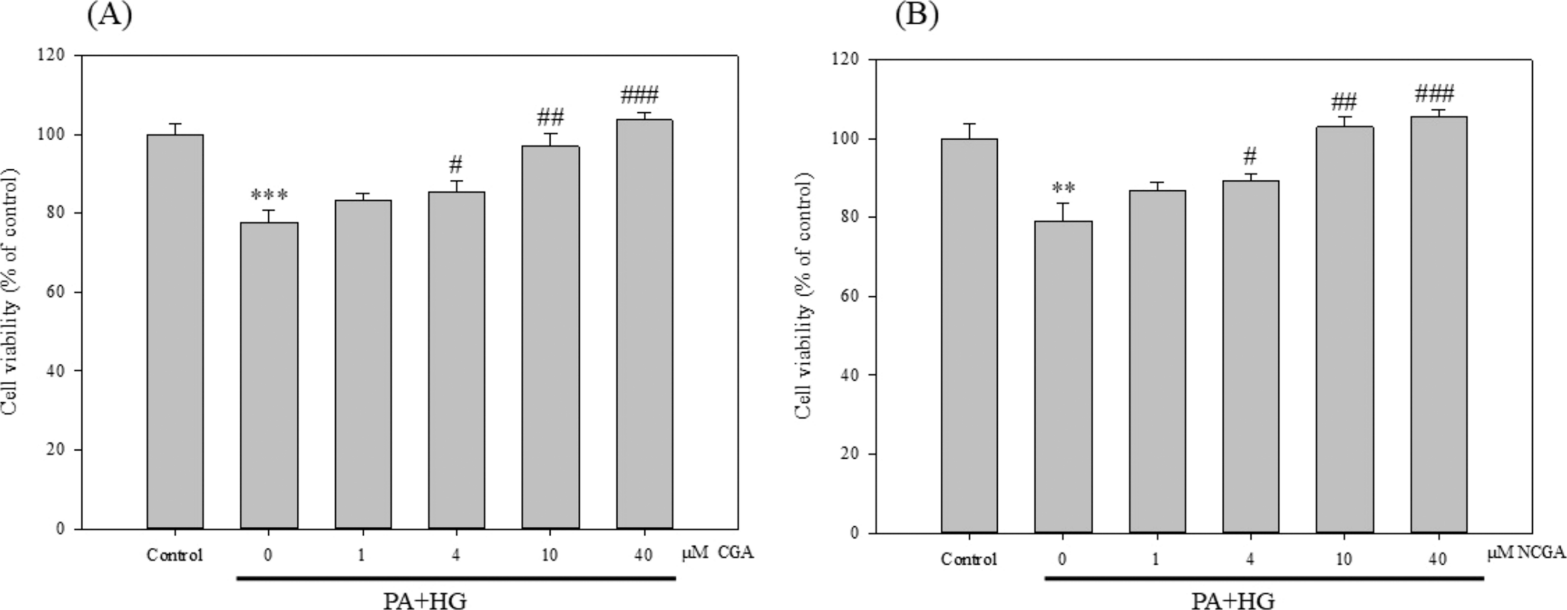
CGA and NCGA mitigate PA- and HG-induced cell death in SH-SY5Y cells. Cells were treated with different concentrations of CGA (A) or NCGA (B). Data from triplicate experiments are expressed as mean ± SD; ** and *** denote *p* < 0.01 and *p* < 0.001 for differences versus the control, respectively, while ^#^, ^##^, and ^###^ denote *p* < 0.05, *p* < 0.01, and *p* < 0.001 for differences with respect to 0 μM CGA or NCGA group, respectively.

Nontoxic doses of CGA and NCGA (≤40 μM) in SH-SY5Y cells were determined via the MTT assay, and these concentrations were used for subsequent experiments. The viability of cells subjected to treatments with PA (0–0.5 mM) or glucose (0–200 mM) for 24 or 48 h declined in a dose-dependent manner (data not shown). In accordance with previous findings of decreased cell viability at 50 mM glucose (HG) or 0.3 mM PA,^21,22^ these concentrations were employed herein to mimic a diabetic environment. The cells were treated with various concentrations of CGA or NCGA (0, 1, 4, 10, and 40 μM) with or without PA and HG for 24 h prior to the MTT assay. The results revealed positive correlation between cell viability and the concentration of CGA/NCGA. Remarkably, treatment with 40 μM CGA/NCGA prevented PA- and HG-induced cell death, restoring the survival rate to that found in the control group (Figure 2). These results reveal the significant neuroprotective potential of CGA and NCGA, warranting further investigations into the underlying mechanism.

### CGA and NCGA Ameliorate PA- and HG-Induced Neuroinflammation in SH-SY5Y Cells

**Figure 3:**
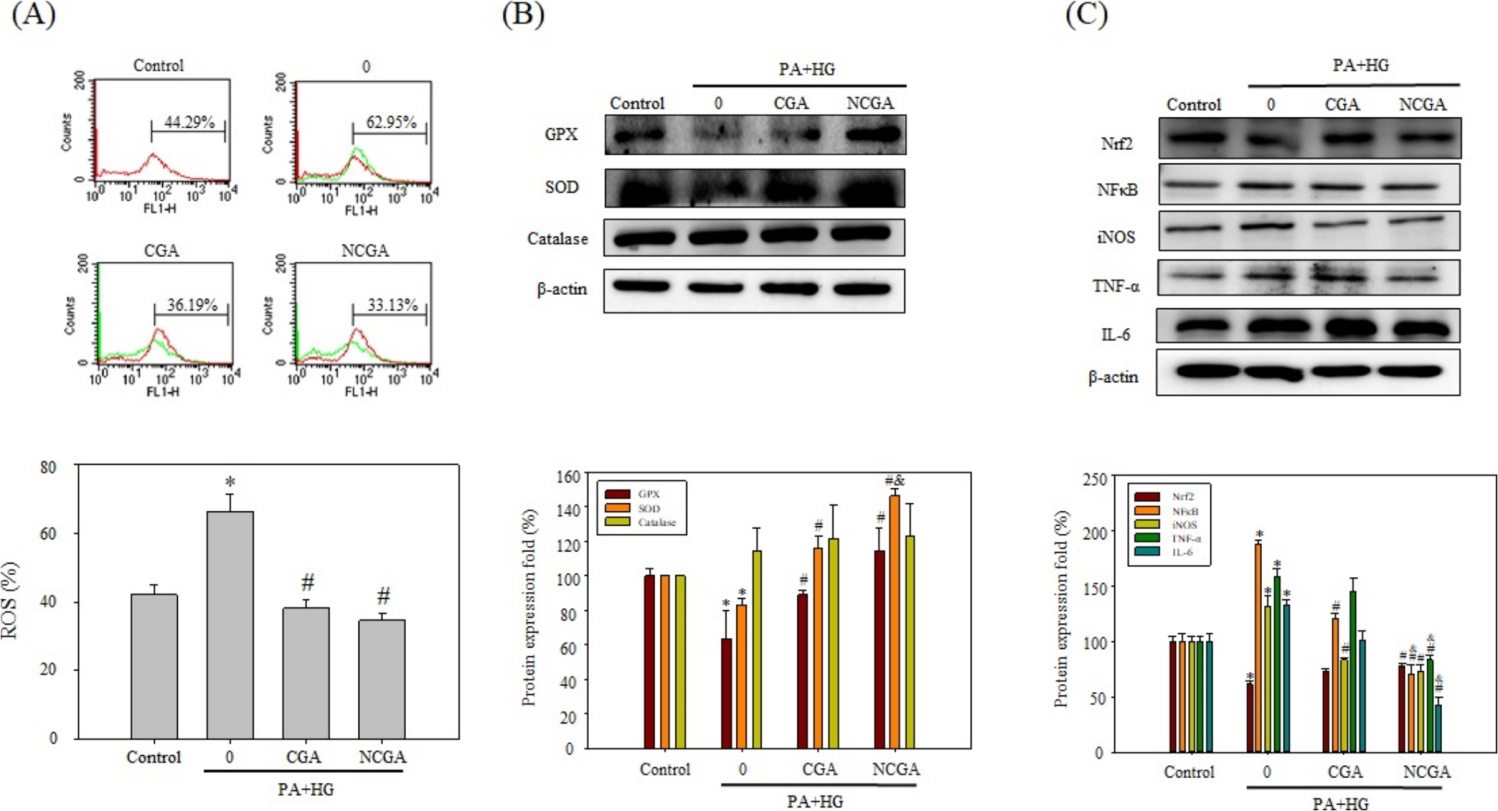
CGA and NCGA mitigate oxidative stress and inflammation in SH-SY5Y cells. (A) ROS generation was measured by flow cytometry analysis following the treatment of SH-SY5Y cells for 24 h. (B, C) Expression of antioxidant enzymes (B) and inflammation-related proteins (C) in SH-S5Y cells treated with PA + HG and CGA or NCGA were determined by Western blot analysis. Data from triplicate experiments have been expressed as mean ± SD; * denotes *p* < 0.05 for differences with respect to the control, ^#^ denotes *p* < 0.05 with respect to 0 μM CGA or NCGA, and ^&^ denotes *p* < 0.05 with respect to the CGA group.

Neuroinflammation caused by oxidative stress can induce neuronal cell death.^23^ To investigate the neuroprotective effects of CGA and NCGA against inflammation-induced damage, the intracellular ROS content in SH-SY5Y cells was measured via flow cytometry. As shown (Figure 3A), the ROS content in the PA + HG group was higher (62.95%) than that of the control group (44.29%), indicating that treatment with PA and HG induced the accumulation of ROS. Conversely, CGA or NCGA treatment resulted in a significant decrease in ROS production in PA- and HG-treated cells, with ROS levels dropping to 36.19 and 33.13%, respectively, indicating the strong antioxidant capacities of these compounds and their potential role in the prevention of neurodegeneration. Subsequently, the expression of key antioxidant enzymes of the cellular antioxidant defense system, including glutathione peroxidase (GPX), superoxide dismutase (SOD), and catalase, was investigated. The expression of GPX and SOD was significantly lower in the PA + HG group (63.44 and 83.12%, respectively) compared with that of the control group (Figure 3B). However, the addition of CGA or NCGA resulted in a remarkable increase in the expression of GPX and SOD. Furthermore, a significant difference in SOD expression between the CGA and NCGA groups indicated the stronger antioxidant capacity of NCGA compared to that of CGA. However, an obvious difference in catalase expression was not detected between the control and treatment groups. These results suggest that CGA and NCGA can protect SH-SY5Y cells subjected to PA + HG treatment from ROS accumulation via the increase in GPX and SOD expression, with NCGA likely to be more potent than CGA.

Studies have shown that the transcription factor Nrf2 plays a crucial role in regulating antioxidant proteins, such as heme oxygenase-1 (HO-1), NAD(P)H quinone dehydrogenase 1 (NQO1), SOD, and GPX, to prevent oxidative stress, neuroinflammation, and neuronal apoptosis.^24^ The results in Figure 3C demonstrate that CGA and NCGA treatment enhanced the expression of the anti-inflammatory factor Nrf2 in PA- and HG-treated cells. Next, the anti-inflammatory activities of CGA and NCGA were investigated by evaluating the expression of key mediators of inflammation. As shown (Figure 3C), the expression of NFκB, inducible nitric oxide synthase (iNOS), TNF-α, and IL-6 was significantly higher in the PA + HG group (188.10, 131.12, 158.23, and 132.52%, respectively) compared to that in the control group. However, CGA treatment attenuated the expression of these proinflammatory mediators to 120.86, 83.71, 145.28, and 100.89%, respectively. Similarly, NCGA treatment notably decreased the expression of these mediators to 70.00, 73.15, 83.45, and 42.47%, respectively. Significant differences in the expression of NFκB, TNF-α, and IL-6 were detected in the NCGA group compared to those in the CGA group, indicating that NCGA may be more effective in reducing neuroinflammation than CGA. In addition, CGA and NCGA treatment enhanced the expression of the anti-inflammatory factor Nrf2 in PA- and HG-treated cells. Overall, both CGA and NCGA can attenuate inflammation, while NCGA treatment exerted a more significant impact on all of the estimated mediators. These results suggest that NCGA may exhibit stronger anti-inflammatory effects via certain pathways compared with those of CGA.

### CGA and NCGA Inhibit PA- and HG-Induced Apoptosis in SH-SY5Y Cells

**Figure 4:**
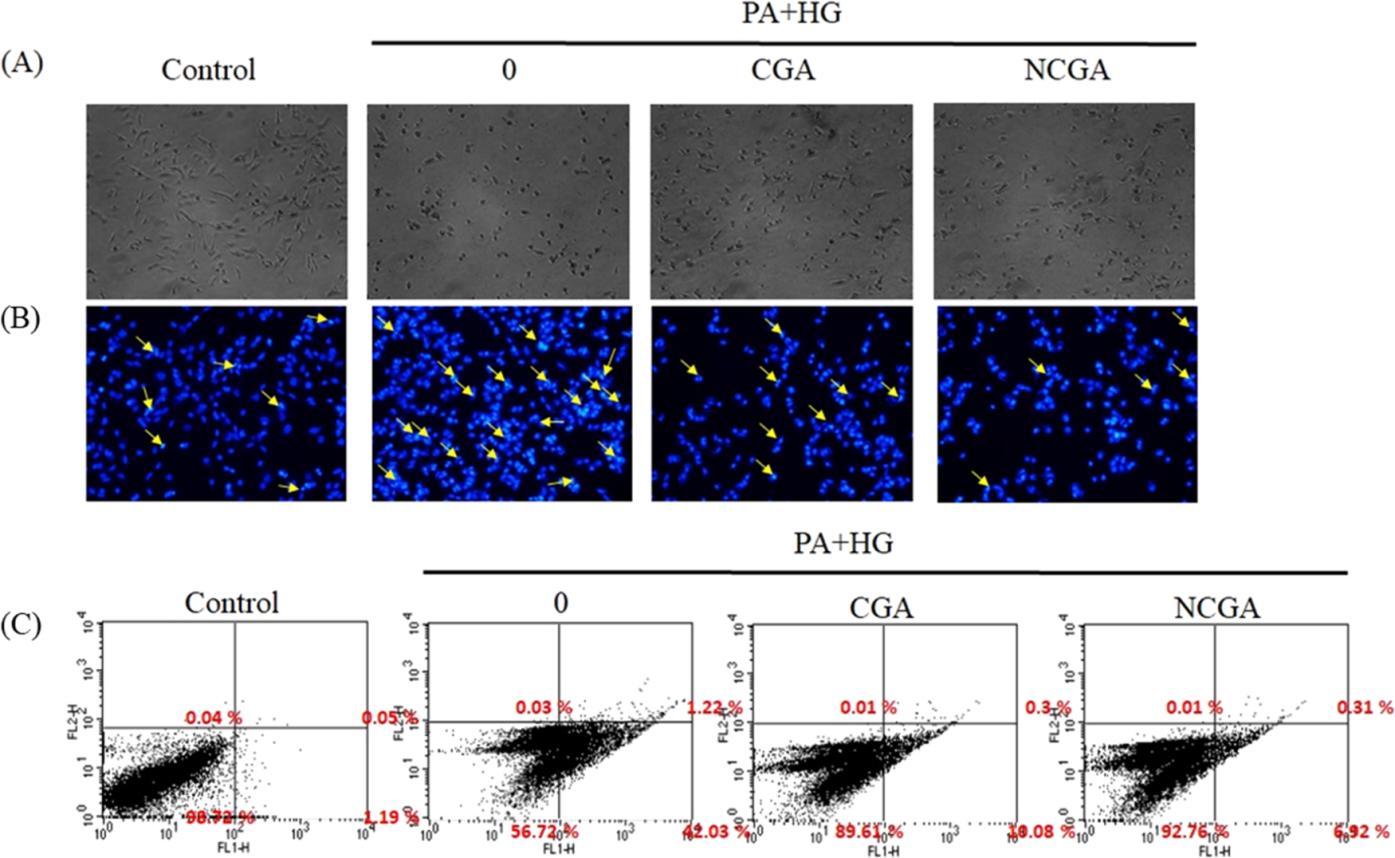
CGA and NCGA afford protection against apoptosis in SH-SY5Y cells. (A) Cell morphology analysis. (B) Visualization of DAPI-stained cells, with yellow arrows indicating nuclear fragmentation, an indicator of apoptosis. (C) Flow cytometry analysis. Cells were stained with Annexin V and PI.

Cellular protrusions found in neural cells aid in receiving and transmitting signals, which are crucial for the normal functioning of neural cells. As shown in Figure 4A, the treatment of SH-SY5Y cells with PA and HG resulted in loss of the normal cellular protrusions and adoption of a small and dense cellular morphology, whereas CGA and NCGA treatment mitigated this effect, resulting in an increased proportion of cells exhibiting normal morphology; of the two compounds, NCGA exhibited a more significant effect. However, treatment with CGA or NCGA failed to completely reverse the neuronal injury caused by PA and HG.

Apoptosis, another important mechanism leading to the death of dopaminergic neurons and a causative factor in PD, also plays a crucial role in neurodegeneration. DAPI staining (Figure 4B) revealed an increase in nuclear fragmentation and the proportion of apoptotic bodies in the PA + HG group compared to those in the control group. Furthermore, the PA + HG group displayed a higher proportion of early apoptotic cells (PI^–^/Annexin^+^; 42.03%) compared to that of the control group (1.19%), suggesting that PA + HG treatment caused early apoptosis in SH-SY5Y cells within 24 h of treatment (Figure 4C). This outcome suggests that PA and HG treatment causes an increase in the apoptosis rate, which may be another mechanism underlying the decrease in cell viability (as evaluated by the MTT assay) associated with the treatment. However, CGA and NCGA treatment reduced the proportion of apoptotic bodies as well as the early apoptotic rates (to 10.08 and 6.92%, respectively). These results indicate that CGA and NCGA considerably inhibit PA- and HG-induced early apoptosis in SH-SY5Y cells, demonstrating their potential to prevent neuronal death in patients with type 2 DM. Interestingly, these results also demonstrate that NCGA is more potent than CGA in reducing the early apoptotic rate, which is consistent with those of the previous experiments.

### CGA and NCGA Modulate Signaling Pathways and Apoptotic Regulators to Mitigate PA- and HG-Induced Cell Death in SH-SY5Y Cells

**Figure 5:**
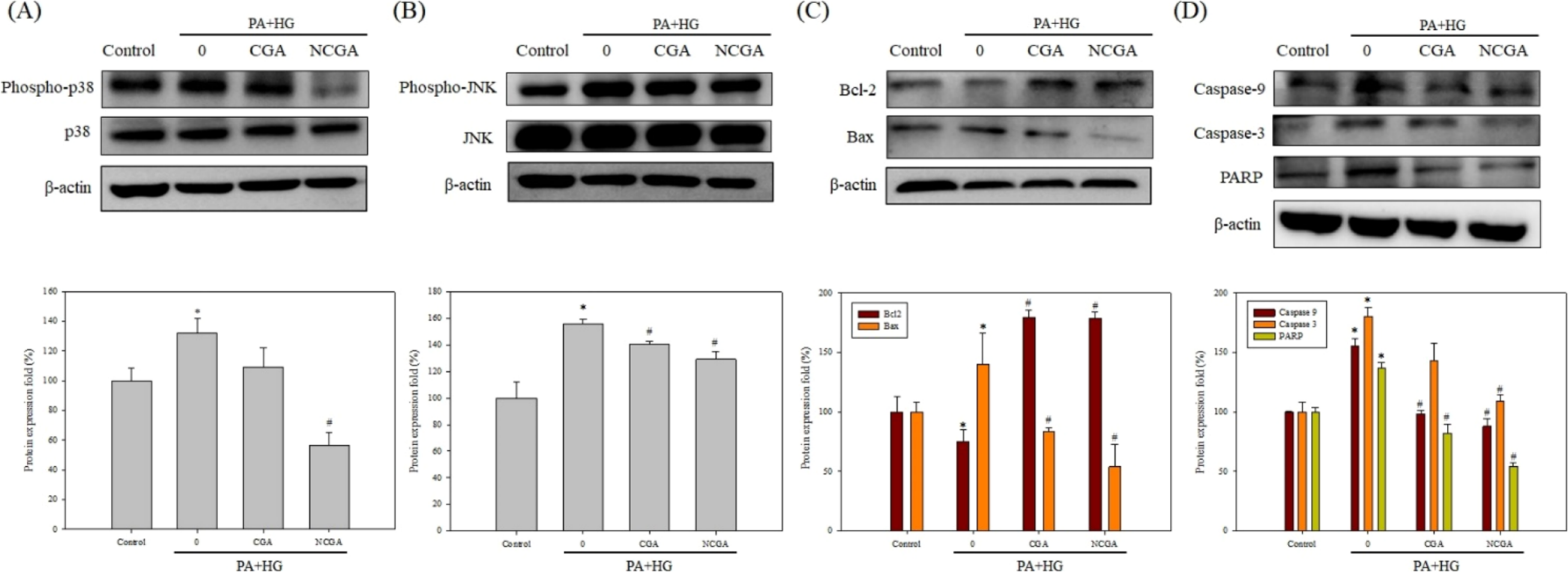
CGA and NCGA treatment decreases the expression of apoptosis-related proteins in SH-SY5Y cells. (A–D) Western blot analysis of p-p38, p38, p-JNK, JNK, Bcl2, Bax, caspase 9, caspase 3, and PARP. Data from triplicate experiments have been expressed as mean ± SD; * denotes *p* < 0.05 for differences with respect to control, and ^#^ denotes *p* < 0.05 with respect to 0 μM CGA or NCGA group.

The mechanism underlying the effect of CGA and NCGA treatment on apoptotic rates was further investigated by Western blot analysis. The significant increase in the expression of phospho-p38 (p-p38)/p38 and phospho-JNK (p-JNK)/JNK in the PA + HG group suggested the activation of the mitogen-activated protein kinase (MAPK) pathway, which may lead to various outcomes such as cell proliferation, differentiation, inflammation, and apoptosis (Figure 5A,B). CGA and NCGA treatments downregulated the MAPK pathway by reducing the expression of both p-JNK/JNK and p-p38/p38, mitigating the increase induced by PA and HG treatment. In addition, lower Bcl2 and higher Bcl2-associated X protein (Bax) expression in the PA + HG group demonstrates that the PA and HG treatment activated the intrinsic pathway of apoptosis in SH-SY5Y cells (Figure 5C). However, both CGA and NCGA mitigated apoptosis by decreasing the expression of Bax and increasing the level of Bcl2, which inhibits the intrinsic pathway of apoptosis. Furthermore, the intrinsic pathway of apoptosis involves the downstream caspases, caspase 9 and caspase 3, and poly(ADP-ribose) polymerase (PARP); the expression of these proteins was therefore analyzed. As shown in Figure 5D, PA and HG treatment increased the expression of all the three proteins, confirming the induction of apoptosis by PA + HG treatment. Further, the expression of caspase 9, caspase 3, and cleaved PARP decreased after treatment with CGA or NCGA, with NCGA exhibiting a better apoptosis-alleviating effect.

### Effect of CGA and NCGA Treatment on Brain-Derived Neurotrophic Factor (BDNF), Aβ, TH, and α-Synuclein in SH-SY5Y Cells

**Figure 6:**
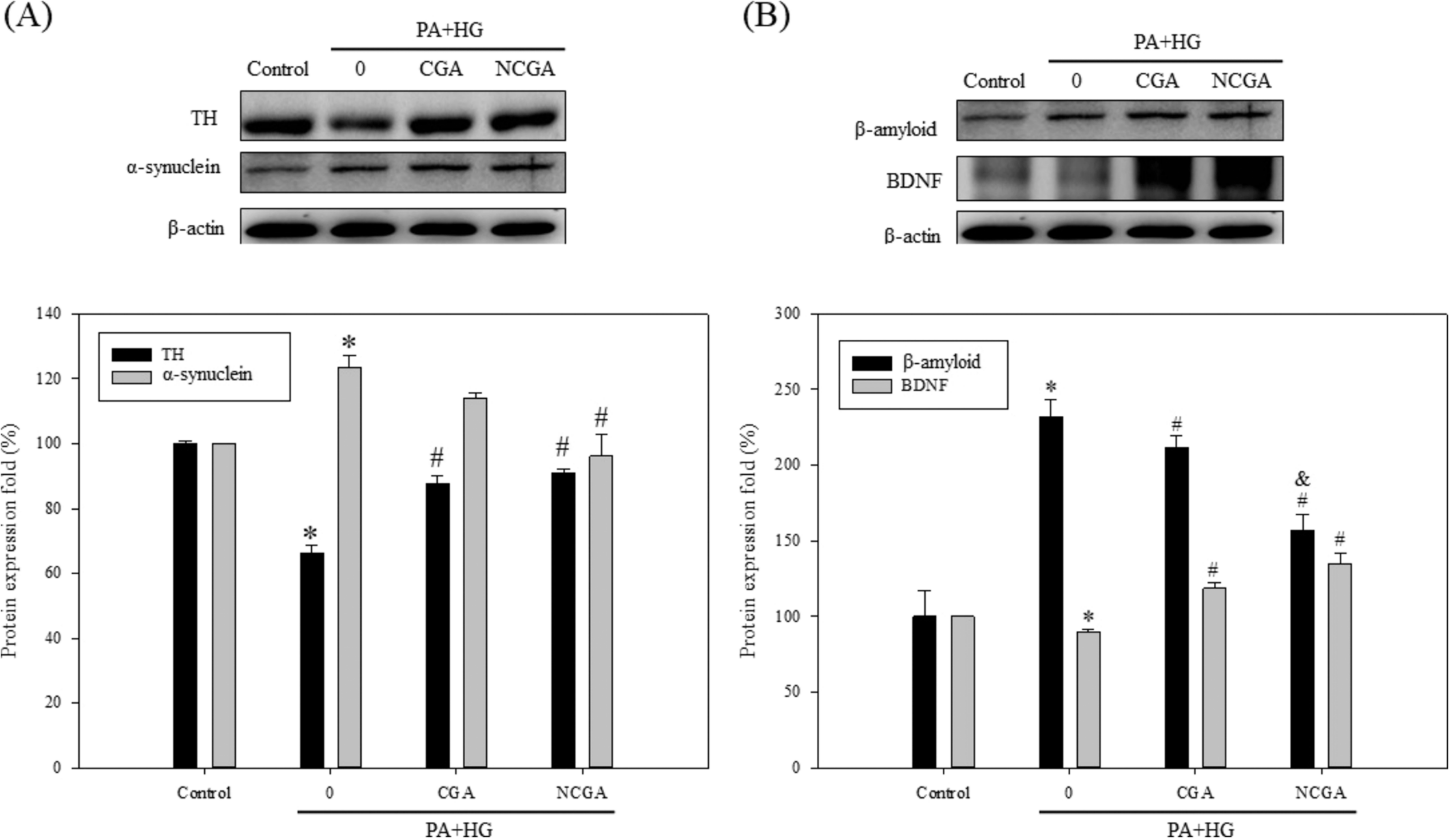
Effect of CGA and NCGA treatment on the expression of molecules associated with neuronal functions in SH-SY5Y cells. (A, B) Western blot analysis to evaluate the expression of TH, α-synuclein, Aβ, and BDNF. Data from triplicate experiments have been expressed as mean ± SD; * denotes *p* < 0.05 for differences with respect to the control, ^#^ denotes *p* < 0.05 with respect to 0 μM CGA or NCGA, and ^&^ denotes *p* < 0.05 with respect to the CGA group.

The modulatory effects of CGA and NCGA treatment on key molecules associated with neuronal development, including TH, α-synuclein, Aβ, and BDNF, were elucidated. As shown (Figure 6A), the SH-SY5Y cells treated with PA and HG expressed lower levels of TH compared with those in the untreated control cells. However, CGA or NCGA treatment increased the expression of TH in SH-SY5Y cells. PA and HG treatment increased the expression of α-synuclein, which was significantly reduced only upon NCGA treatment. Additionally, the expression of Aβ increased substantially (to 232.34%) in the PA + HG group compared to that in the control group (set as 100%), while CGA and NCGA treatments mitigated the increase in its expression to 211.40 and 156.82%, respectively (Figure 6B). BDNF plays a crucial role in the development, survival, and functioning of neurons. The expression of BDNF was found to decrease (to 90.08%) in the PA + HG group, while CGA and NCGA treatment reversed the trend, increasing the expression to 118.50 and 135.22%, respectively. Subsequently, the neuroprotective effects of CGA and NCGA were evaluated in the db/db mouse model maintained on an HFD.

### Validation of the db/db Mouse Model of Diabetes

After a 12-week feeding period, serum samples were collected from the mice to evaluate lipid and glucose levels in the blood, as well as liver and kidney functions in the mice. A notable tendency toward DM was observed in the db/db mice, as evidenced by the elevated levels of triglycerides, cholesterol, HDL-C, ALT, CRE, BUN, UA, HbA1c, and insulin (Table 1). The aberrant patterns of serum parameters observed in the HFD group are consistent with the characteristics of DM. However, supplementation with MLE led to a decrease in all of these parameters. Collectively, these findings indicate that db/db mice effectively manifest the genetic traits of DM, making them a suitable model for studying this condition.

**Table 1:** Effect of MLE on the Serum Biochemical Parameters Compared with db and HFD Groups in a DM Mouse Model^a^

	control	db/db	HFD	MLE
TG (mg/dL)	78.33 ± 13.61	167.0 ± 31.43	350.0 ± 45.57^b^	161.33 ± 14.15^c^
cholesterol (mg/dL)	62.67 ± 16.07	211.0 ± 32.92	450.0 ± 32.08^b^	354.33 ± 26.69^c^
LDL-C (mg/dL)	15.33 ± 3.51	19.33 ± 8.74	167.0 ± 4.58^b^	118.0 ± 2.0^c^
HDL-C (mg/dL)	36.0 ± 9.54	99.67 ± 25.03	144.0 ± 10.15^b^	110.67 ± 7.51^c^
AST (U/L)	74.0 ± 8.54	84.33 ± 10.60	259.0 ± 42.44^b^	144.0 ± 36.51^c^
ALT (U/L)	34.67 ± 5.51	63.0 ± 27.40	566.33 ± 69.64^b^	339.67 ± 48.43^c^
UA (mg/dL)	3.07 ± 0.15	5.90 ± 1.23	9.87 ± 0.32^b^	7.17 ± 0.51^c^
CRE (mg/dL)	0.47 ± 0.06	0.60 ± 0.10	0.80 ± 0.10^b^	0.63 ± 0.06^c^
BUN (mg/dL)	16.4 ± 2.98	22.67 ± 1.21	31.80 ± 2.34^b^	15.47 ± 1.32^c^
HbA1c (%)	4.0 ± 0.17	8.90 ± 0.98	9.30 ± 0.96	8.73 ± 0.51
insulin (pg/L)	0.98 ± 0.10	5.30 ± 0.53	7.97 ± 0.94^b^	5.53 ± 0.51^c^

a The mice were divided into four groups: control (C57BL/6 fed with a normal diet), db (db/db mice fed with a normal diet), HFD (db/db mice fed with a high-fat diet), MLE (db/db mice fed with a high-fat diet and additional MLE). Data are expressed as mean ± SD for 3 mice.

b *p* < 0.05 vs db.

c *p* < 0.05 vs HFD.

### Effect of MLE Supplementation on the Corpus Striatum and Substantia Nigra of HFD-Treated db/db Mice

**Figure 7:**
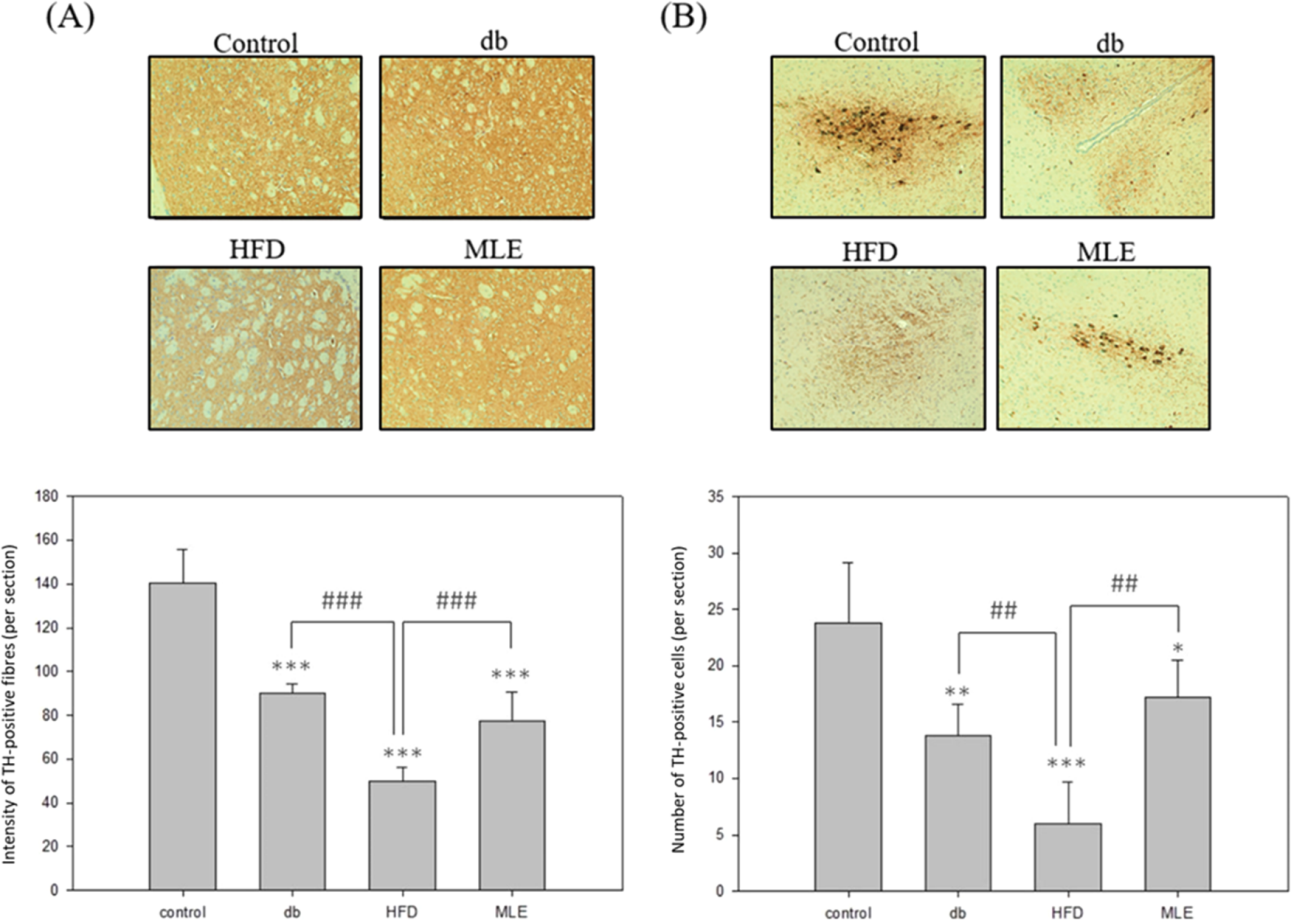
MLE supplementation enhances the expression of TH in the corpus striatum and substantia nigra of the db/db mouse model of diabetes. IHC analysis to evaluate TH expression in (A) corpus striatum and (B) substantia nigra of the db/db mice. Data from triplicate experiments have been expressed as mean ± SD; *, **, and *** denote *p* < 0.05, *p* < 0.01, and *p* < 0.001 for differences with respect to the control group, respectively, while ^##^ and ^###^ denote *p* < 0.01 and p < 0.001 for differences with respect to the HFD group, respectively.

**Figure 8:**
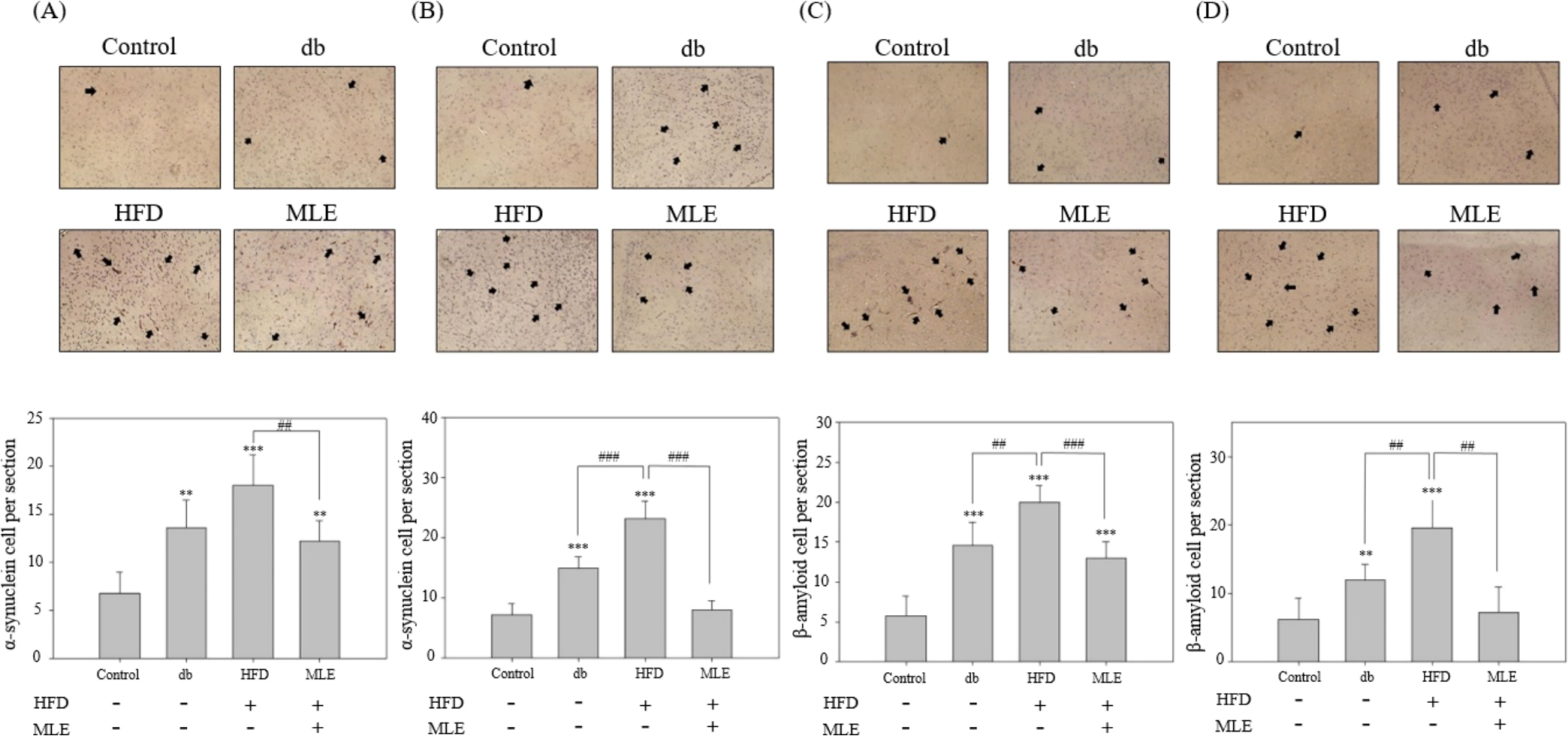
MLE supplementation reduces the expression of α-synuclein and Aβ in the corpus striatum and substantia nigra of the db/db mouse model of diabetes. IHC analysis to evaluate the expression of (A, B) α-synuclein and (C, D) Aβ in the corpus striatum (A, C) and substantia nigra (B, D). Data from triplicate experiments have been expressed as mean ± SD; ** and *** denote *p* < 0.01 and *p* < 0.001 for differences with respect to the control, while ^##^ and ^###^ denote *p* < 0.01 and *p* < 0.001 for differences with respect to the HFD group.

To evaluate whether HFD induces neurodegeneration in the brains of db/db mice and assess the potential of MLE to ameliorate this condition, IHC was conducted to analyze the expression of key markers associated with neurodegeneration including TH, α-synuclein, and Aβ in the corpus striatum and substantia nigra of the mice. Reduced expression of TH, which is indicative of functional loss, was observed in the db/db group maintained on HFD, which was significantly improved upon MLE treatment (Figure 7A,B). These findings suggest that MLE affords protection to the dopaminergic neurons in db/db mice maintained on HFD. Furthermore, the expression of α-synuclein in the corpus striatum and substantia nigra of the db/db group was 2-fold higher than that in the control group. HFD further exacerbated this condition by further increasing the expression of α-synuclein (Figure 8A,B). However, MLE supplementation significantly decreased α-synuclein expression in the corpus striatum compared to that in the db/db mice maintained on an HFD (Figure 8A). Notably, MLE supplementation reduced the expression of α-synuclein in the substantia nigra to the extent found in that of the control group (Figure 8B), indicating the potential of MLE to greatly mitigate α-synuclein-mediated damage in the substantia nigra. Similarly, higher expression of Aβ was observed in the HFD group, which was reduced upon MLE supplementation, particularly in the substantia nigra (Figure 8C,D). These results suggest that MLE supplementation may effectively prevent damage caused by misfolded proteins in the substantia nigra while only alleviating the negative effects caused by type 2 DM and HFD in the corpus striatum.

## Discussion

The prevalence of motor coordination impairments resulting from the loss of dopaminergic neurons is increasing worldwide. Recent evidence suggests that chronic metabolic disorders, such as diabetes, may substantially increase the risk of losing these essential neurons, which are critical for voluntary movement and motor function. This has generated considerable interest in exploring exogenous compounds capable of mitigating the loss of dopaminergic neurons and enhancing motor function. The current study investigates the neuroprotective effects of CGA and NCGA, two major compounds found in mulberry leaves, against HG- and high lipid-induced toxicity in neuronal cells as well as their therapeutic potential in a db/db mouse model of diabetes-mediated neurodegeneration.

The neuroprotective effects of numerous phenolic acids, including ferulic, caffeic, and CGA, have been well established. For instance, ferulic acid has been shown to significantly attenuate memory impairment, reduce neuronal apoptosis in the hippocampus, decrease oxidative stress, and afford protection against injury caused by acute ischemic stroke via the inhibition of excitotoxicity, inflammation, and apoptosis.^25^ Caffeic acid exhibits the ability to inhibit neuronal apoptosis and afford protection against neuroinflammation, underscoring its therapeutic potential in neurodegenerative diseases. Additionally, caffeic acid has been shown to alleviate HFD-induced memory impairment in rats with hyperinsulinemia by enhancing hippocampal neurogenesis, further underscoring its neuroprotective effects. The various beneficial effects of CGA on neuronal health are well recognized; however, its potential to protect neurons from damage induced by HG- and high-lipid environments that are commonly associated with diabetes has not been entirely elucidated. Herein, we focus on CGA and its derivative, NCGA, as key compounds of interest due to their neuroprotective properties. This is the first study to demonstrate the neuroprotective effects of CGA and NCGA in SH-SY5Y cells subjected to PA- and HG-induced diabetes-like conditions and elucidate the underlying mechanism involving antioxidant, anti-inflammatory, and antiapoptotic pathways. The treatment of SH-SY5Y cells with CGA and NCGA resulted in a significant reduction in ROS levels. The underlying mechanisms were further explored by Western blot analysis, which revealed that both compounds mitigate inflammation by modulating the expression of mediators of inflammation. Additionally, the neuroprotective effects of these compounds are associated with the inhibition of apoptosis and, thereby, increased cell survival. These *in vitro* findings were further supported by *in vivo* experiments, which revealed decreased expression of Aβ and α-synuclein alongside increased expression of TH in both the substantia nigra and corpus striatum of the db/db mice. These results underscore the therapeutic potential of CGA and NCGA for the treatment of patients with diabetes, who are at risk of developing neurodegenerative diseases.

Oxidative stress results from an imbalance between ROS production and antioxidant defenses and contributes significantly to neuronal injury and death in neurodegenerative diseases. Neuroinflammation, primarily mediated by the microglia and astrocytes, occurs in response to neuronal damage and results in the release of proinflammatory cytokines, which exacerbate oxidative stress. This inflammatory response can further promote neuronal apoptosis, which is a crucial process for tissue homeostasis. This creates a vicious cycle wherein oxidative stress activates neuroinflammatory pathways, which in turn leads to further generation of ROS and the induction of apoptosis, resulting in increased neuronal loss. This complex interaction is a key mechanism underlying the progression of neurodegenerative diseases such as AD and PD.^26,27^ Flow cytometry analysis revealed that CGA and NCGA exhibit antioxidant activities and reduce ROS levels in SH-SY5Y cells. This demonstrates their antioxidant properties and reflects their potential to inhibit subsequent pathological processes, such as inflammation and apoptosis. Similar results were reported in other studies that investigated the effects of different phenolic acids on SH-SY5Y cells.^28,29^ The results obtained herein also demonstrate that CGA and NCGA can attenuate inflammation by reducing the expression of proinflammatory cytokines. Notably, NCGA was found to exhibit superior anti-inflammatory effects compared to those of CGA, with the latter significantly affecting the expression of only NFκB and iNOS compared to the corresponding levels in the PA + HG group. By contrast, NCGA impacted the expression of all inflammation-related markers that were evaluated herein, including Nrf2, NFκB, iNOS, TNF-α, and IL-6. This is the first study to highlight the potentially greater efficacy of NCGA over that of CGA.

AD is the most common type of neurodegenerative disease worldwide; however, disease pathogenesis is not entirely understood yet. One of the most prominent hypotheses is that of the deposition of extracellular Aβ in the brain to form the Aβ plaque, a process preceded by oxidative stress and neuroinflammation.^30,31^ The results obtained herein reveal that CGA and NCGA can mitigate oxidative stress and inflammation that occur before the deposition of Aβ. Western blot analysis (Figure 6B), however, revealed that the expression of Aβ did not decrease to the levels found in the control group, suggesting that the damage to SH-SY5Y cells resulting from the PA + HG treatment can persist even after supplementation with CGA/NCGA. Moreover, these findings underscore the complexity of the pathology of AD, revealing that addressing oxidative stress and neuroinflammation, while crucial, may not be sufficient to entirely prevent the accumulation of Aβ. This highlights the need for a multifaceted therapeutic approach that targets various aspects of the disease. The inability of CGA and NCGA to completely reduce Aβ levels suggests that additional strategies, potentially involving the modulation of Aβ clearance mechanisms or inhibition of its production, may be necessary. Furthermore, elucidating the optimal timing and duration of CGA and NCGA treatment can be vital, as early intervention may yield more favorable outcomes and prevent the progression of neurodegenerative changes.^32^ Overall, the results obtained herein support the potential application of CGA and NCGA as complementary agents in a broader therapeutic strategy aimed at mitigating the onset and progression of AD. Further investigations in preclinical and clinical settings are warranted.

The estimation of BDNF levels is essential for understanding the neuroprotective effects of CGA and NCGA, as BDNF is a key neurotrophin that supports neuronal survival and improves neurotransmission as well as motor performance.^33^ Western blot analysis revealed a significant increase in BDNF expression following treatment with CGA and NCGA. This effect may be mediated via the activation of the tropomycin receptor kinase B (TrkB) receptor signaling pathway, which is known to promote neuronal survival and reduce apoptosis.^34,35^ Additionally, the antioxidant properties of these compounds may mitigate oxidative stress, further facilitating BDNF signaling.^36^ This is in line with the results of a previous study, which showed that quercetin, a flavonoid with potent antioxidant effects, can reduce apoptosis in a rat model of focal cerebral ischemia.^37^ While these findings are promising, additional studies are required for validating the underlying mechanisms and determining the optimal dosage of CGA and NCGA.

Interestingly, the antioxidant, anti-inflammatory, and antiapoptotic activities of the relatively less-studied NCGA appear to be superior to those of CGA. NCGA treatment was found to increase the expression of SOD to a greater extent than that of CGA, suggestive of an enhanced antioxidant capacity. Additionally, NCGA treatment decreased the expression of NFκB, TNF-α, and IL-6, the key markers of inflammation, in a more effective manner than that of CGA, demonstrating the potent anti-inflammatory properties of NCGA. Furthermore, NCGA treatment reduced the expression of p-p38/p38 and activity of caspase 3 to a greater degree, implying stronger inhibition of the proapoptotic pathways. Notably, NCGA treatment reduced the levels of α-synuclein and Aβ, two proteins implicated in the pathogenesis of PD and AD, respectively, in a more significant manner compared with that of CGA. These results suggest that NCGA may be more potent in modulating certain key pathways involved in neurodegeneration. However, despite these differences, overall cell viability was not significantly different between the CGA and NCGA treatment groups in the current study. While these findings highlight the potential therapeutic benefits of NCGA, further research is required to investigate the underlying mechanisms of action in detail and to compare its efficacy with that of CGA in greater detail using various neuronal models and experimental paradigms.

The associations between DM and dopaminergic cell death as well as the resulting impairment in motor functions highlight the critical need for interventions aimed at protecting dopaminergic neurons.^38^ Previous research has confirmed that MLE contains many antioxidant components^12,13^ and thereby, has the potential to prevent the death of dopaminergic neurons. To assess the effects of MLE supplementation on neuronal health and functionality, the expression of TH, α-synuclein, and Aβ was evaluated. A quantification of these biomarkers can provide insights into whether MLE can effectively restore and/or maintain the normal functioning of dopaminergic neurons. The current study employed C57BL/6JNarl and db/db mice as animal models. The mice were fed the respective diets for 12 weeks and then sacrificed; the corpus striatum and substantia nigra were then isolated and analyzed via IHC. This is the first investigation of the effects of MLE on the brain of mice and revealed that MLE supplementation can enhance the expression of TH while reducing those of α-synuclein and Aβ, which is consistent with the findings from *in vitro* experiments. However, MLE supplementation failed to restore TH expression to the levels observed in the control group. Additionally, although MLE supplementation significantly reduced the expression of α-synuclein and Aβ in the substantia nigra to levels comparable to those in the control group, it failed to achieve similar reductions in the corpus striatum. This discrepancy between the effects of MLE on the substantia nigra and corpus striatum can potentially be attributed to several mechanisms. First, the regional differences in the permeability of the blood–brain barrier between the substantia nigra and corpus striatum may have allowed more effective penetration and accumulation of the active compounds of MLE within the substantia nigra.^39^ Second, the substantia nigra may exhibit earlier responses to the treatment, while the corpus striatum may require longer treatment durations to exhibit significant changes. Further research in the future on these potential mechanisms may aid in optimizing treatment protocols to facilitate the effective targeting of both the substantia nigra and the corpus striatum.

The current study highlights the neuroprotective potential of CGA and NCGA in promoting neuronal health. Both of the compounds demonstrated significant antioxidant, anti-inflammatory, and antiapoptotic activities, with NCGA exhibiting great efficacy in enhancing the expression of SOD and reducing those of the proinflammatory cytokines such as NFκB, TNF-α, and IL-6. Notably, this is the first study to present evidence of the neuroprotective effects of NCGA on SH-SY5Y cells under diabetes-like conditions. Additionally, MLE supplementation in animal models resulted in reduced levels of α-synuclein and Aβ concomitantly with an increased expression of TH, indicating the protective effects of MLE against neurodegeneration. Despite these promising results, further research investigating the long-term effects, optimal dosages, and mechanisms of action of CGA and NCGA are warranted. Furthermore, future studies should focus on the therapeutic potential of these compounds in more complex *in vivo* models. Overall, this research contributes to the consideration of CGA and NCGA as potential neuroprotective agents in various neurodegenerative diseases. In summary, this study contributes to our understanding of the potential value of CGA and NCGA application to achieve improvement in neurodegenerative diseases.

## Data Availability

No new data were created or analyzed in this study. Data sharing is not applicable to this article.

## Notes

**Institutional Review Board Statement** The study was conducted in accordance with the Institutional Animal Care and Use Committee (IACUC) of the Chung Shan Medical University, Taichung, Taiwan (Approval Number 2025).

## Notes

**Informed Consent Statement** Not applicable.

## References

[ref1] Mukhtar Y.; Galalain A.; Yunusa U. A modern overview on diabetes mellitus: a chronic endocrine disorder. Eur. J. Biol. 2020, 5 (2), 1–14. 10.47672/ejb.409.

[ref2] Belenichev I.; Aliyeva O.; Popazova O.; Bukhtiyarova N. Molecular and biochemical mechanisms of diabetic encephalopathy. Acta Biochim. Pol. 2023, 70 (4), 751–760. 10.18388/abp.2020_6953. 37991083

[ref3] Galizzi G.; Di Carlo M. Mitochondrial DNA and Inflammation in Alzheimer’s Disease. Curr. Issues Mol. Biol. 2023, 45 (11), 8586–8606. 10.3390/cimb45110540. 37998717 PMC10670154

[ref4] Sulzer D.; Cragg S. J.; Rice M. E. Striatal dopamine neurotransmission: regulation of release and uptake. Basal Ganglia 2016, 6 (3), 123–148. 10.1016/j.baga.2016.02.001. 27141430 PMC4850498

[ref5] Dodson P. D.; Dreyer J. K.; Jennings K. A.; Syed E. C.; Wade-Martins R.; Cragg S. J.; Bolam J. P.; Magill P. J. Representation of spontaneous movement by dopaminergic neurons is cell-type selective and disrupted in parkinsonism. Proc. Natl. Acad. Sci. U.S.A. 2016, 113 (15), E2180–E2188. 10.1073/pnas.1515941113. 27001837 PMC4839395

[ref6] Ni A.; Ernst C. Evidence that substantia nigra pars compacta dopaminergic neurons are selectively vulnerable to oxidative stress because they are highly metabolically active. Front. Cell. Neurosci. 2022, 16, 826193 10.3389/fncel.2022.826193. 35308118 PMC8931026

[ref7] Dionísio P.; Amaral J. D.; Rodrigues C. M. P. Oxidative stress and regulated cell death in Parkinson’s disease. Ageing Res. Rev. 2021, 67, 101263 10.1016/j.arr.2021.101263. 33540042

[ref8] Bai R.; Guo J.; Ye X. Y.; Xie Y.; Xie T. Oxidative stress: The core pathogenesis and mechanism of Alzheimer’s disease. Ageing Res. Rev. 2022, 77, 101619 10.1016/j.arr.2022.101619. 35395415

[ref9] Pajares M.; A I. R.; Manda G.; Boscá L.; Cuadrado A. Inflammation in Parkinson’s Disease: Mechanisms and Therapeutic Implications. Cells 2020, 9 (7), 1687 10.3390/cells9071687. 32674367 PMC7408280

[ref10] Dhiman S.; Kumar V.; Mehta C. M.; Gat Y.; Kaur S. Bioactive compounds, health benefits and utilisation of Morus spp.– a comprehensive review. J. Hortic. Sci. Biotechnol. 2020, 95 (1), 8–18. 10.1080/14620316.2019.1644969.

[ref11] Chan E. W.-C.; Lye P.-Y.; Wong S.-K. Phytochemistry, pharmacology, and clinical trials of Morus alba. Chin. J. Nat. Med. 2016, 14 (1), 17–30. 10.3724/SP.J.1009.2016.00017. 26850343

[ref12] Chan E. W. C.; Wong S. K.; Tangah J.; Inoue T.; Chan H. T. Phenolic constituents and anticancer properties of Morus alba (white mulberry) leaves. J. Integr. Med. 2020, 18 (3), 189–195. 10.1016/j.joim.2020.02.006. 32115383

[ref13] Lee Y.-J.; Hsu J.-D.; Lin W.-L.; Kao S.-H.; Wang C.-J. Upregulation of caveolin-1 by mulberry leaf extract and its major components, chlorogenic acid derivatives, attenuates alcoholic steatohepatitis via inhibition of oxidative stress. Food Funct. 2017, 8 (1), 397–405. 10.1039/C6FO01539E. 28074952

[ref14] Yan Y.; Li Q.; Shen L.; Guo K.; Zhou X. Chlorogenic acid improves glucose tolerance, lipid metabolism, inflammation and microbiota composition in diabetic db/db mice. Front. Endocrinol. 2022, 13, 1042044 10.3389/fendo.2022.1042044. PMC9714618 36465648

[ref15] Ye X.; Li J.; Gao Z.; Wang D.; Wang H.; Wu J. Chlorogenic Acid Inhibits Lipid Deposition by Regulating the Enterohepatic FXR-FGF15 Pathway. BioMed Res. Int. 2022, 2022 (1), 4919153 10.1155/2022/4919153. 35257010 PMC8897747

[ref16] Kurita S.; Kashiwagi T.; Ebisu T.; Shimamura T.; Ukeda H. Identification of neochlorogenic acid as the predominant antioxidant in Polygonum cuspidatum leaves. Ital. J. Food Sci. 2016, 28 (1), 25–31.

[ref17] Kozyra M.; Komsta Ł.; Wojtanowski K. Analysis of phenolic compounds and antioxidant activity of methanolic extracts from inflorescences of Carduus sp. Phytochem. Lett. 2019, 31, 256–262. 10.1016/j.phytol.2019.04.012.

[ref18] Mirza F.; Lorenzo J.; Drissi H.; Lee F. Y.; Soung D. Y. Dried plum alleviates symptoms of inflammatory arthritis in TNF transgenic mice. J. Nutr. Biochem. 2018, 52, 54–61. 10.1016/j.jnutbio.2017.10.002. 29149648

[ref19] Park S. Y.; Jin M. L.; Yi E. H.; Kim Y.; Park G. Neochlorogenic acid inhibits against LPS-activated inflammatory responses through up-regulation of Nrf2/HO-1 and involving AMPK pathway. Environ. Toxicol. Pharmacol. 2018, 62, 1–10. 10.1016/j.etap.2018.06.001. 29908432

[ref20] Tsai M.-C.; Wang C.-C.; Tsai I.-N.; Yu M.-H.; Yang M.-Y.; Lee Y.-J.; Chan K.-C.; Wang C.-J. Improving the Effects of Mulberry Leaves and Neochlorogenic Acid on Glucotoxicity-Induced Hepatic Steatosis in High Fat Diet Treated db/db Mice. J. Agric. Food Chem. 2024, 72 (12), 6339–6346. 10.1021/acs.jafc.3c09033. 38488910 PMC10979445

[ref21] Liu Y.; Sun L.; Ma Y.; Wei B.; Gao M.; Shang L. High glucose and bupivacaine-induced cytotoxicity is mediated by enhanced apoptosis and impaired autophagy via the PERK-ATF4-CHOP and IRE1-TRAF2 signaling pathways. Mol. Med. Rep. 2019, 20 (3), 2832–2842. 10.3892/mmr.2019.10524. 31524237 PMC6691238

[ref22] Ng Y. W.; Say Y. H. Palmitic acid induces neurotoxicity and gliatoxicity in SH-SY5Y human neuroblastoma and T98G human glioblastoma cells. PeerJ 2018, 6, e4696 10.7717/peerj.4696. 29713567 PMC5924683

[ref23] Karvandi M. S.; Hesari F. S.; Aref A. R.; Mahdavi M. The neuroprotective effects of targeting key factors of neuronal cell death in neurodegenerative diseases: The role of ER stress, oxidative stress, and neuroinflammation. Front. Cell. Neurosci. 2023, 17, 1105247 10.3389/fncel.2023.1105247. 36950516 PMC10025411

[ref24] Ngo V.; Duennwald M. L. Nrf2 and oxidative stress: A general overview of mechanisms and implications in human disease. Antioxidants 2022, 11 (12), 2345 10.3390/antiox11122345. 36552553 PMC9774434

[ref25] Szwajgier D.; Borowiec K.; Pustelniak K. The Neuroprotective Effects of Phenolic Acids: Molecular Mechanism of Action. Nutrients 2017, 9 (5), 477 10.3390/nu9050477. 28489058 PMC5452207

[ref26] Teleanu D. M.; Niculescu A.-G.; Lungu I. I.; Radu C. I.; Vladâcenco O.; Roza E.; Costăchescu B.; Grumezescu A. M.; Teleanu R. I. An overview of oxidative stress, neuroinflammation, and neurodegenerative diseases. Int. J. Mol. Sci. 2022, 23 (11), 5938 10.3390/ijms23115938. 35682615 PMC9180653

[ref27] Karvandi M. S.; Hesari F. S.; Aref A. R.; Mahdavi M. The neuroprotective effects of targeting key factors of neuronal cell death in neurodegenerative diseases: The role of ER stress, oxidative stress, and neuroinflammation. Front. Cell. Neurosci. 2023, 17, 1105247 10.3389/fncel.2023.1105247. 36950516 PMC10025411

[ref28] Gay N. H.; Phopin K.; Suwanjang W.; Songtawee N.; Ruankham W.; Wongchitrat P.; Prachayasittikul S.; Prachayasittikul V. Neuroprotective Effects of Phenolic and Carboxylic Acids on Oxidative Stress-Induced Toxicity in Human Neuroblastoma SH-SY5Y Cells. Neurochem. Res. 2018, 43 (3), 619–636. 10.1007/s11064-017-2463-x. 29417471

[ref29] González-Sarrías A.; Núñez-Sánchez M. Á.; Tomás-Barberán F. A.; Espín J. C. Neuroprotective Effects of Bioavailable Polyphenol-Derived Metabolites against Oxidative Stress-Induced Cytotoxicity in Human Neuroblastoma SH-SY5Y Cells. J. Agric. Food Chem. 2017, 65 (4), 752–758. 10.1021/acs.jafc.6b04538. 28142243

[ref30] Roy R. G.; Mandal P. K.; Maroon J. C. Oxidative Stress Occurs Prior to Amyloid Aβ Plaque Formation and Tau Phosphorylation in Alzheimer’s Disease: Role of Glutathione and Metal Ions. ACS Chem. Neurosci. 2023, 14 (17), 2944–2954. 10.1021/acschemneuro.3c00486. 37561556 PMC10485904

[ref31] Foret M. K.; Orciani C.; Welikovitch L. A.; Huang C.; Cuello A. C.; Carmo S. D. Early oxidative stress and DNA damage in Aβ-burdened hippocampal neurons in an Alzheimer’s-like transgenic rat model. Commun. Biol. 2024, 7 (1), 861 10.1038/s42003-024-06552-4. 39004677 PMC11247100

[ref32] Barnett J. H.; Lewis L.; Blackwell A. D.; Taylor M. Early intervention in Alzheimer’s disease: a health economic study of the effects of diagnostic timing. BMC Neurol. 2014, 14 (1), 101 10.1186/1471-2377-14-101. 24885474 PMC4032565

[ref33] Palasz E.; Wysocka A.; Gasiorowska A.; Chalimoniuk M.; Niewiadomski W.; Niewiadomska G. BDNF as a Promising Therapeutic Agent in Parkinson’s Disease. Int. J. Mol. Sci. 2020, 21 (3), 1170 10.3390/ijms21031170. 32050617 PMC7037114

[ref34] Gupta V. K.; You Y.; Gupta V. B.; Klistorner A.; Graham S. L. TrkB Receptor Signalling: Implications in Neurodegenerative, Psychiatric and Proliferative Disorders. Int. J. Mol. Sci. 2013, 14 (5), 10122–10142. 10.3390/ijms140510122. 23670594 PMC3676832

[ref35] Jaboin J.; Kim C. J.; Kaplan D. R.; Thiele C. J. Brain-derived neurotrophic factor activation of TrkB protects neuroblastoma cells from chemotherapy-induced apoptosis via phosphatidylinositol 3′-kinase pathway. Cancer Res. 2002, 62 (22), 6756–6763. 12438277

[ref36] Wu A.; Ying Z.; Gomez-Pinilla F. The interplay between oxidative stress and brain-derived neurotrophic factor modulates the outcome of a saturated fat diet on synaptic plasticity and cognition. Eur. J. Neurosci. 2004, 19 (7), 1699–1707. 10.1111/j.1460-9568.2004.03246.x. 15078544

[ref37] Yao R.-Q.; Qi D.-S.; Yu H.-L.; Liu J.; Yang L.-H.; Wu X.-X. Quercetin Attenuates Cell Apoptosis in Focal Cerebral Ischemia Rat Brain Via Activation of BDNF–TrkB–PI3K/Akt Signaling Pathway. Neurochem. Res. 2012, 37 (12), 2777–2786. 10.1007/s11064-012-0871-5. 22936120

[ref38] Labandeira C. M.; Fraga-Bau A.; Ron D. A.; Alvarez-Rodriguez E.; Vicente-Alba P.; Lago-Garma J.; Rodriguez-Perez A. I. Parkinson’s disease and diabetes mellitus: common mechanisms and treatment repurposing. Neural Regener. Res. 2022, 17 (8), 1652–1658. 10.4103/1673-5374.332122. PMC8820685 35017411

[ref39] Ha I. H.; Lim C.; Kim Y.; Moon Y.; Han S. H.; Moon W. J. Regional Differences in Blood-Brain Barrier Permeability in Cognitively Normal Elderly Subjects: A Dynamic Contrast-Enhanced MRI-Based Study. Korean J. Radiol. 2021, 22 (7), 1152–1162. 10.3348/kjr.2020.0816. 33739632 PMC8236362

